# *E. coli* Histidine Triad Nucleotide Binding Protein 1 (ecHinT) Is a Catalytic Regulator of D-Alanine Dehydrogenase (DadA) Activity *In Vivo*

**DOI:** 10.1371/journal.pone.0020897

**Published:** 2011-07-06

**Authors:** Sanaa Bardaweel, Brahma Ghosh, Tsui-Fen Chou, Michael J. Sadowsky, Carston R. Wagner

**Affiliations:** 1 Department of Medicinal Chemistry, University of Minnesota, Minneapolis, Minnesota, United States of America; 2 Department of Soil, Water and Climate and the Biotechnology Institute, University of Minnesota, St. Paul, Minnesota, United States of America; Texas A&M University, United States of America

## Abstract

Histidine triad nucleotide binding proteins (Hints) are highly conserved members of the histidine triad (HIT) protein superfamily. Hints comprise the most ancient branch of this superfamily and can be found in Archaea, Bacteria, and Eukaryota. Prokaryotic genomes, including a wide diversity of both Gram-negative and Gram-positive bacteria, typically have one Hint gene encoded by *hinT* (*ycfF* in *E. coli*). Despite their ubiquity, the foundational reason for the wide-spread conservation of Hints across all kingdoms of life remains a mystery. In this study, we used a combination of phenotypic screening and complementation analyses with wild-type and *hinT* knock-out *Escherichia coli* strains to show that catalytically active ecHinT is required in *E. coli* for growth on D-alanine as a sole carbon source. We demonstrate that the expression of catalytically active ecHinT is essential for the activity of the enzyme D-alanine dehydrogenase (DadA) (equivalent to D-amino acid oxidase in eukaryotes), a necessary component of the D-alanine catabolic pathway. Site-directed mutagenesis studies revealed that catalytically active C-terminal mutants of ecHinT are unable to activate DadA activity. In addition, we have designed and synthesized the first cell-permeable inhibitor of ecHinT and demonstrated that the wild-type *E. coli* treated with the inhibitor exhibited the same phenotype observed for the *hinT* knock-out strain. These results reveal that the catalytic activity and structure of ecHinT is essential for DadA function and therefore alanine metabolism in E. coli. Moreover, they provide the first biochemical evidence linking the catalytic activity of this ubiquitous protein to the biological function of Hints in *Escherichia coli*.

## Introduction

Histidine triad nucleotide binding proteins (Hints) are conserved from bacteria to humans [1]. While prokaryotes typically encode only one Hint gene, eukaryotes generally contain two to three different Hint genes [2]. The physiological role of Hints in mammalian cells is just now being delineated. Two hybrid screening experiments revealed that Hint1 interacts with Cdk7, the catalytic subunit of the cyclin dependent kinase activation complex Cdk7-cyclin H-MAT1 [3]. However, Hint1 mouse knock-out studies indicated that Hint1 is not required for Cdk7 function [4]. The human Hint1 (hHint1) has also been shown to directly interact with human Pontin and Reptin in the TCF-β-catenin transcription complex [5]. It has been reported that hHint1 might be a tumor suppressor [6], [7]. Like other tumor suppressor proteins, Hint1 is associated with pro-apoptotic properties [8]. More recently, human Hint1 (hHint1) has been reported to participate in the development of hepatic ischemia reperfusion injury [9]. In addition, recent studies have shown that hHint1 is involved in the regulation of postsynaptic dopamine transmission [10]. Recently, we reported that hHint1 and *E.coli* Hint (ecHinT) hydrolyze lysine-AMP generated by bacterial and human LysRS, suggesting that Hints have a specific role in regulating LysRS [11].

*E. coli* Hint (encoded by *hinT*) has high sequence similarity to hHint1 (ecHinT is 48% identical to hHint1 at the amino acid level) and has been shown to form stable potential protein-protein interactions with six proteins: a putative oxidoreductase and formate dehydrogenase (b1501), the heat shock protein 70 (Hsp70), the β-subunit of DNA polymerase III (*dnaN*), a membrane-bound lytic murein transglycosylase D (*dniR*), ET-Tu elongation factor (*tufA*), and a putative synthetase (*yjhH*) [12]. In addition, the HinT from *Mycoplasma* has been shown to interact with two membrane proteins (P60 and P80) [13], [14]. With regard to the importance of Hint1 catalytic activity, yeast strains constructed with a catalytically deficient mutant of the ortholog, Hnt1, failed to grow on galactose at elevated temperatures, suggesting that the catalytic activity of Hnt1 is required for the phenotype [15]. Nevertheless, the physiological and biochemical basis for the relationship of Hints to these interactions and associated phenotypes, as well as the foundational reason for their wide-spread conservation across all three kingdoms of life remains enigmatic.

Crystal structure studies of hHint1 and, recently, ecHinT have revealed that both proteins are homodimers containing an active-site with four conserved histidines [16], [17]. While similar, close inspection of the two structures revealed little sequence similarity between their C-termini. In contrast to hHint1, the longer C-terminus of ecHinT was found to adopt eight different conformations in the unit cell [17]. Chimeric domain swap mutants, in which the C-termini of both ecHinT and hHint1 have been switched, have demonstrated the importance of the C-termini on model substrate specificity [18]. Moreover, deletion mutagenesis studies have shown that the loss of just three C-terminal side-chains abolishes the ability of ecHinT to hydrolyze LysRS-generated lysine-AMP, while having only a modest effect on the catalytic efficiency of the enzyme with model substrates [17]. Although catalytic insights of Hint activity have been garnered from these studies, a defined biochemical rationale for the function of Hints in general, and ecHinT in particular, has remained elusive.

Phenotype characterization is an essential approach for understanding structure-function relationships among a variety of biological systems. While several advanced and comprehensive technologies have been developed to sequence and identify functions of genes and their products, and assign them to particular metabolic pathways, the function of many genes in most organisms that have been sequenced to date remains unknown. For example, although *E. coli* is considered to be amongst the most genetically characterized microorganisms, about 30–40% of its genes have unknown function [19]. In an effort to determine the function of many of these “unknown” genes, a library of single gene knock-out mutants of all nonessential *E. coli* K-12 genes has been generated [20]. The metabolic profiles of many of these knock-out mutants have been characterized using Phenotype MicroArrays (PM) that allow testing of a large number of cellular phenotypes in 96-well microplates [21]. Based on the same redox chemistry, Biolog™ phenotypic screening plates have been developed as a simplified universal reporter of metabolism in a single bacterium.

Given the high sequence similarity between hHint1 and ecHinT, we hypothesized that determining the function of Hint in *E. coli* may reveal a conserved biochemical and physiological role for Hints in general. In *E. coli* K-12, *in silico* analysis indicates that *hinT* is located in an apparent operon consisting of the *hinT(ycfF)-ycfL-ycfM-ycfN(thiK)-ycfO(nagZ)-ycfP* genes. In the study reported here, we investigated the role of *hinT* in *E. coli* by carrying out Biolog™ phenotypic metabolic analyses with single gene deletion mutants. In addition, we investigated the role of ecHinT catalysis and structure on the observed ecHinT phenotype with a combination of site-directed mutagenesis and chemical biological studies. Our results show that ecHinT catalytic activity and the C-terminal domain are required for *E. coli* to grow on D-alanine as a sole carbon and energy source by the regulation of D-amino acid dehydrogenase activity. Taken together, our results demonstrate that ecHinT plays an essential role in the regulation of the fate of alanine in cellular compartments and thus links for the first time the catalytic activity and structure of a Hint protein with a bacterial physiological function.

## Methods

### Bacterial strains, media, and growth conditions

The bacterial strains used in this study were obtained from the *E. coli* Genetic Stock Center at Yale University and are listed in Table S1. All strains were received as glycerol stocks on filters, sub-cultured twice on Luria–Bertani (LB) agar medium [22], and incubated at 37°C for 48 h before testing. Liquid cultures were grown aerobically in LB medium supplemented with kanamycin (50 µg/ml) at 37°C, with shaking, for 18 h. Strains were stored as 10% glycerol stocks at −80°C.

### PCR verification of mutants

The mutations in gene knock-out mutants were verified using the polymerase chain reaction (PCR) technique. Primers sequences for each PCR reaction are listed in Table S2. DNA from freshly isolated colonies served as templates for PCR and reactions were initiated following a “hot start” protocol at 95°C. The first PCR reaction was used to confirm the expected size of *hinT* operon with a single gene deletion and insertion of a *kan* resistance gene. The second PCR reaction confirmed the junction points of the *kan* resistance gene in each knock-out mutant. PCR conditions were as follows: 94°C for 2 min followed by 30 cycles of 94°C for 30 s, 55°C for 30 s, and 72°C for 2 min.

### Verification of *hinT* deletion mutant by loss of activity

The turnover rates of the fluorogenic substrate tryptamine 5′-adenosine phosphoramidate (TpAd) by ecHinT protein in cell-free lysates of wild-type *E. coli* BW25113 and the *hinT* deletion mutant (Δ*hinT*) was determined using a previously described phosphoramidase assay [23]. The excitation wavelength was 280 nm and fluorescence emission was measured at 360 nm. All kinetic assays were performed in duplicate at 25°C. The substrate hydrolysis rate was determined by measuring fluorescence increase following addition of 5–10 µl of lysates (8 ng total protein).

### Phenotype analysis using Biolog™ GN2-MicroPlates

Phenotypic analyses of *hinT* operon mutants containing single gene deletions were determined using Biolog™ GN2-MicroPlates (Biolog Inc., Hayward, CA). Mutants were inoculated onto R2A agar plates containing, per liter, 0.5 g casamine acids, 0.5 g of D-glucose, 0.3 g sodium pyruvate, 0.3 g K_2_HPO_4_, 0.05 g MgSO_4_·7H_2_O, 0.5 g proteose peptone, 0.5 g soluble starch, 0.5 g yeast extract, and 15 g agar and incubated overnight at 37°C. Cells were swabbed from the surface of the agar plate, suspended in GN/GP-IF inoculating fluid (Biolog Inc., Hayward , CA) to a final OD_600 nm_ of 0.7, and inoculated into the GN2-MicroPlate (150 µl per well). The microplates were incubated at 30°C for 24 h, and absorbance values at 540 nm and 630 nm were determined using an EL808 Ultra Microplate reader (Bio-Tek Instruments Inc., Winooski, VT).

### Carbon source utilization assays

Overnight cultures of wild-type and mutant *E. coli* strains were grown at 37°C, with shaking at 200 rpm, in LB medium supplemented with kanamycin (50 µg/ml) as needed. Cell pellets were obtained by centrifugation at 6000× g at 4°C for 10 min. Pellets were washed twice and re-suspended in minimal medium containing (per liter): 6.8 g Na_2_HPO_4_, 3.0 g KH_2_PO_4_, 0.5 g NaCl, 1.0 g NH_4_Cl, 0.001 g CaC1_2_, 0.001 g MgSO_4_, and 0.004 g thiamine, (pH 7.0). Carbon sources, D,L-alanine, glycerol, or glucose, were added, after autoclaving the medium, to a final concentration of 20 mM when needed.

### Induction of D-amino acid dehydrogenase activity and enzyme assays

Induction of D-amino acid dehydrogenase in wild-type and mutant strains was achieved by adding 100 mM D,L-alanine to minimal medium containing 10% (vol/vol) glycerol as the sole carbon source. Cultures were grown at 37°C for 24 h prior to collection of cell pellets. D-amino acid dehydrogenase activity was measured by following the reduction of 2,6-dichlorophenol-indophenol (DCPIP) at 600 nm as previously described [24]. Reaction mixtures contained 50 mM potassium phosphate buffer (pH 7.0), 10 mM KCN, 0.5 mM phenazine methosulfate, 10 mM DCPIP, and 100 mM of D,L-alanine. The reduction of absorbance at 600 nm was measured for 5 min using Varian Cary 50 UV-visible spectrophotometer (Palo Alto. CA, USA). The amount of DCPIP reduced was determined using a molar extinction coefficient of 21,500. All assays were produced in triplicate, and rates were determined from the first 2 min of activity. Activity is expressed as nmoles DCPIP reduced/min/mg protein, and rates between replicates varied by <5%.

### Preparation of membrane fractions

Wild-type and mutant strains were grown to late exponential phase (OD_600 nm_ = 0.9–1.1) in minimal medium containing 100 mM D,L-alanine and 10% (vol/vol) glycerol, harvested by centrifugation at 10, 000× g, and washed once in 0.85% NaCl. The pellets were stored at −80°C. Cell pellets were thawed, suspended in MOPS Buffer (10 mM MOPS, (pH 7.5), 20 mM MgCl_2_, and 10% glycerol) and passed twice through a French Pressure Cell at 11,000 psi. Cell lysates were centrifuged for 10 min at 14,500× g and the resulting supernatant was centrifuged for 80 min at 37,000× g in a Beckman Ultracentrifuge. The membrane fractions were obtained as described previously [25]. Aliquots of membrane fractions were stored at −80°C and used only once after thawing.

### Reverse transcription-PCR

Total RNA was isolated from *E. coli* strains using Qiagen RNeasy minikit (Qiagen, Valencia, CA) according to the manufacturer's protocols. RNA concentration was determined spectrophotometrically at 260 nm and RNA quality was confirmed by electrophoresis of 1 µg of total RNA from each preparation on a 1.2% denatured agarose gel. The following primers were used for RT-PCR analysis: LP 5′GTTTCGATAACCGCATTCGT3′ and RP 5′CCGGTATTCAGCCACAGATT3′. The RT-PCR conditions were adapted from Qiagen OneStep RT-PCR Kit handbook and were as follows: 50°C for 30 min , 95°C for 15 min followed by 30 cycles of 94°C for 0.5 min, 50°C for 0.5 min, 72°C for 0.5 min followed by a single 10-min cycle at 72°C for extension.

### Site-directed mutagenesis

All *hinT* mutants were generated from *E. coli hinT*-pSGA02, the expression vector harboring the gene encoding wild-type *hinT*, by using the Quick-Change mutagenesis kit (Stratagene, Santa Clara, CA) following the manufacturer's protocol. Mutagenesis of ecHinT and hHint1 to produce *ec*/Hs and Hs/*ec* chimeric proteins was described previously [18].

### Phenotype complementation studies

The preparation of competent cells was achieved using a Z-Competent *E. coli* Transformation Kit and Buffer Set (Zymo Research Corporation, Orange, CA). The Δ*hinT* mutant was transformed with 20 ng of plasmid DNA encoding either the wild-type *hinT*, H101A, H101G, Δ114–119, Δ117–119, Hs/*ec* chimera or *ec*/Hs chimera. Cultures were grown in LB medium, induced with IPTG (500 µM) when the culture reached OD_600 nm_ = 0.4–0.7, and incubated for an additional 10 h. Cell pellets were obtained by centrifugation at 6000× g for 10 min at 4°C, washed twice in M9 minimal medium [22], and re-suspended in 200 µl of the same medium. A 10–20 µl aliquot of the cell suspension was inoculated into M9 medium supplemented with 20 mM D,L-alanine or glucose, with ampicillin (100 µg/ml) and kanamycin (50 µg/ml), to an initial OD_600 nm_ = 0.06–0.08. Cells were incubated at 37°C and OD_600 nm_ was measured after for 36–40 h of growth.

### General synthetic procedures and materials

All reagents were purchased from commercial vendors and used without further purification. Anhydrous pyridine was used from previously unopened SureSeal or AcrosSeal bottles. Analytical thin layer chromatorgraphy (TLC) was performed on 0.25 mm precoated Merck silica gel (SiO_2_) 60 F254. Column chromatography was performed on Purasil 60A silica gel, 230–400 mesh (Whatman). ^1^H and ^31^P NMR were recorded on a Varian Mercury-300 or a Varian MR 400 spectrometer. Chemical shifts are reported in ppm relative to residual dimethyl sulfoxide (DMSO-d6) peak. High-resolution mass spectrometry (HRMS) data were obtained on a Biotof II (Bruker) ESI-MS spectrometer.

### Synthesis of 2′,3′-Isopropylidine-5′-O-(4-chlorophenoxy)carbonyl guanosine (Guanosine -5′-carbonate acetonide, 1)

Guanosine-2′,3′- acetonide (0.231 mmol) was dissolved in anhydrous pyridine (7 ml) in a 2-neck round-bottomed flask equipped with an Ar. inlet and a microsyringe. The stirred solution was chilled over a dry ice-acetone bath and 4-chlorophenyl chloroformate (0.278 mmol, 1.20 eq) was added dropwise under Ar. The cooling bath was removed and the solution was slowly allowed to return to room temperature. Stirring was continued until thin layer chromatographic and ESI-MS analyses showed that all of the starting material had disappeared (after approximately 4 h). Pyridine was evaporated *in vacuo* by co-distillation with n-heptane and the crude solid obtained was purified by chromatography on SiO_2_ gel to isolate the guanosine-5′-carbonate as a while solid with a 90% yield. The ^1^H NMR spectrum was (DMSO-d6): 1.30 (s, 3H, -CH_3_), 1.56 (s, 3H, -CH_3_), 4.28–4.58 (m, 3H, C2′+C3′+C4′-H), 5.22 (s, 2H, 5′-CH_2_), 6.22 (s, 1H, C1′-H), 6.58 (s, 2H, NH_2_), 7.22 (d, 2H, -Ph), 7.50 (d, 2H, -Ph), 7.86 (s, 1H, C8-H), 10.74 (s, 1H, N1-H) ppm.

### Synthesis of 2′,3′-Isopropylidine-5′-O-[(3-indolyl)-1-ethyl]carbamoyl guanosine (Guanosine -5′-carbamate acetonide, 2)

A mixture of guanosine-5′-carbonate (0.255 mmol) and tryptamine (0.640 mmol, 2.5 eq) in anhydrous THF (10 ml) was heated to 60°C (oil bath) under Ar. and stirred at that temperature until TLC showed complete consumption of the starting material (4 h). The solvent was removed by rotary evaporation and the residue chromatographed on SiO_2_ gel using CH_2_Cl_2_∶MeOH∶H_2_O (5∶2∶0.25) to give the title carbamate as white amorphous solid (yield 90%). The ^1^H NMR spectrum was (DMSO-d6): 1.32 (s, 3H, -CH_3_), 1.58 (s, 3H, -CH_3_), 2.80 (t, 2H, -CH_2_-), 3.24 (q, 2H, -*CH2*NHCO 4.00–4.30 (m, 3H, 5′-CH_2_+C4′-H), 5.10 (d, 1H, 5′-C3′-H), 5.24 (d, 1H, C2′-H), 6.00 (s, 1H, C1′-H), 6.58 (s, 2H, NH_2_), 6.96 (t, 1H, indole), 7.12 (t, 1H, -indole), 7.18 (s, 1H, indole), 7.36 (d, 1H, indole), 7.40 (t, 1H, -NH), 7.50 (d, 1H, indole), 7.84 (s, 1H, C8-H), 10.72 (s, 1H, N1-H), 10.82 (s, 1H, NH-indole) ppm.

### Synthesis of 5′-O-[(3-indolyl)-1-ethyl]carbamoyl guanosine (Guanosine-5′- tryptamine carbamate, TpGc)

The above 2′, 3′acetonide-protected guanosine carbamate (100 mg) was dissolved in TFA∶H_2_O (4∶1, 5 ml) and stirred at room temperature until TLC and ESI-MS showed no starting material left (60 min). The mixture was stripped to dryness and the residue purified by SiO_2_ gel chromatography using CH_2_Cl_2_∶MeOH∶H_2_O (5∶3∶0.5) as eluant. The relevant UV-active fractions were collected and concentrated to afford the final compound as a white solid (yield 40%). The ^1^H NMR spectrum was (DMSO-d6): 2.38 (t, 2H, -CH_2_-), 3.17 (q, 2H, -CH2NHCO), 4.23–4.33 (m, 2H, 5′-CH_2_), 4.56 (t, 1H, C4′-H), 5.14 (d, 1H, C3′-H), 5.39 (d, 1H, C2′-H), 6.10 (s, 1H, C1′-H), 6.38 (s, 2H, NH_2_), 6.96 (t, 1H, indole), 7.15 (t, 1H, -indole), 7.21 (s, 1H, indole), 7.32 (d, 1H, indole), 7.40 (t, 1H, -NH), 7.48 (d, 1H, indole), 7.80 (s, 1H, C8-H), 10.65 (s, 1H, N1-H), 10.78 (s, 1H, NH-indole) ppm [OHs exchanging with H_2_O in NMR solvent].

## Results

### Operon structure of genes associated with *hinT*

In *E. coli* K-12 strains, the *hinT* gene is located adjacent to *ycfL*, *ycfM*, *ycfN*(*thiK*), *ycfO*(*nagZ*), and *ycfP*. PCR analyses were performed to determine if the deletion/insertion mutations occurred in frame without affecting the operon structure. Results in Figure S1 show that the operon structure is still intact after deletion of each gene and insertion of a *kan* resistant gene. Further evidence of *hinT* deletion was obtained by measuring the phosphoramidase activity in cell-free lysates. Figure S2 shows that the *hinT* deletion mutant (Δ*hinT*) did not have appreciable phosphoramidase activity that is associated with the expression of *hinT* in wild-type *E. coli* BW25113.

### Phenotype of *ecHinT*

**Figure 1:**
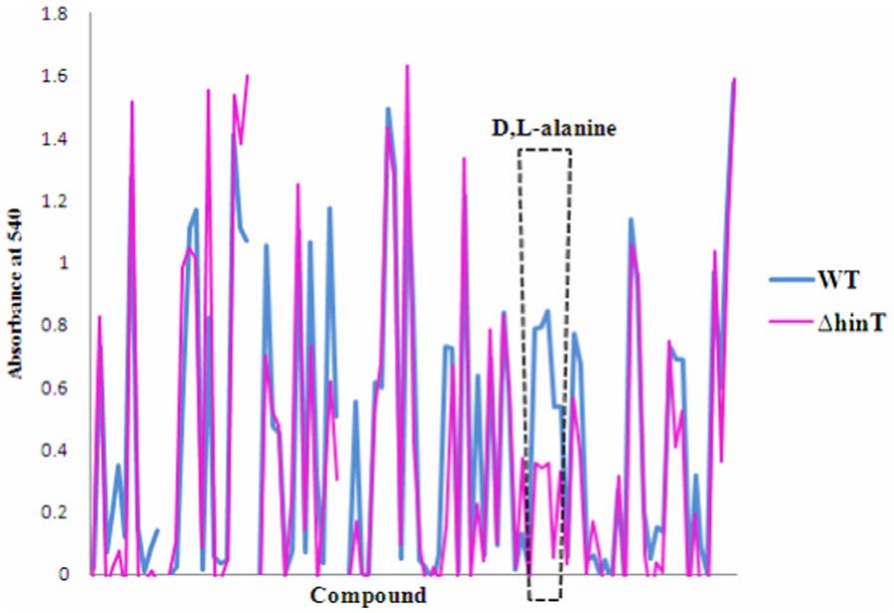
Summary of metabolic fingerprints. Dataset of the GN2 plate assay of wild-type *E. coli* BW25113 and Δ*hinT* mutant strains was processed as described in experimental procedures. The overlay represents the difference between the ability of the two strains to use 95 carbon sources. This is a representative overlay of three independent experiments.

**Figure 2:**
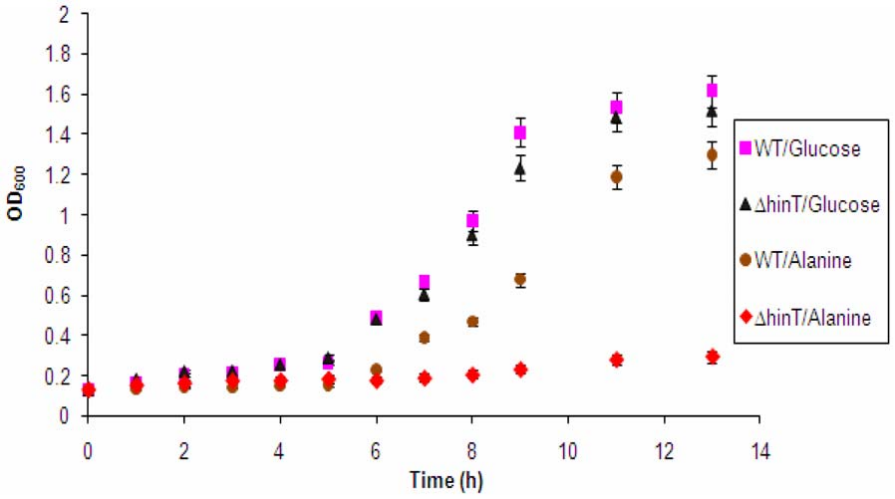
Bacterial growth curves of wild-type *E. coli* BW25113 and Δ*hinT* mutant in M9 medium in the presence of either 20 mM glucose or D,L-alanine. Measurements were done in duplicate and variants were less than 5% between two independent cultures.

To ascertain the role of ecHinT in microbial metabolism, we obtained a series of mutants from the Keio collection containing deletions in the *hinT* (*ycfF*), *ycfL*, *ycfM*, *ycfN*(*thiK*), *ycfO*(*nagZ*), and *ycfP* genes. Biolog™ plates were used for phenotypic screening to estimate the differential metabolic potential between wild-type *E. coli* BW25113 and the *hinT* deletion mutant (Δ*hinT*). Results from Biolog™ substrate utilization studies (Fig. 1) indicated that metabolic profiles of wild-type *E. coli* BW25113 and the Δ*hinT* mutant were highly similar, with the exception of the utilization of D- and L-alanine. The initial screening results were verified and confirmed by culturing wild-type and mutant strains in minimal media containing D,L-alanine (Fig. 2). These results were highly reproducible, even when a starvation phase was introduced prior to culturing.

To further define the role of genes in the apparent *hinT* operon in alanine metabolism, *E. coli* strains from the Keio collection with verified deletions in *ycfL* (JW1090), *ycfM* (JW5157), *ycfN* (JW1092), *ycfO* (JW1093), and *ycfP* (JW5158) were evaluated for carbon source utilization. Compared to the wild-type BW25113 strain, none of the tested mutants showed significant differences in their ability to catabolize alanine when tested using Biolog™ plates and test tube cultures (data not shown).

### D-amino acid transcription and activity testing

**Figure 3:**
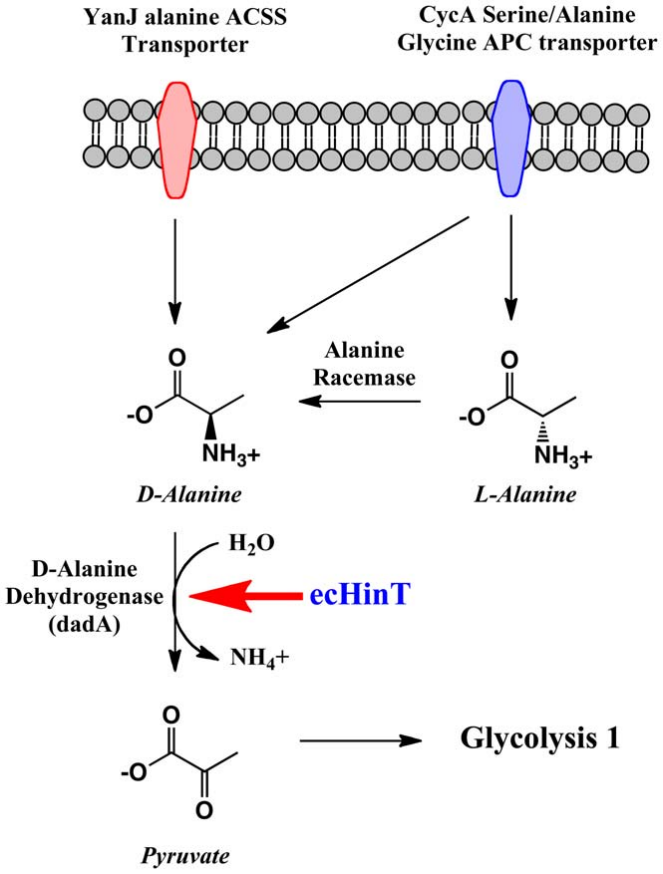
Alanine transport and metabolism in *E. coli* and the proposed role of ecHinT.

**Figure 4:**
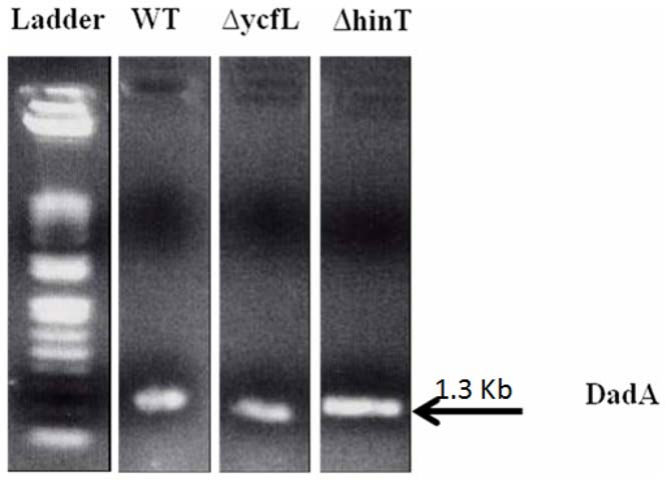
RT-PCR was performed with equivalent amounts of mRNA (100 ng) obtained from wild-type *E. coli* BW25113, Δ*hinT*, and Δ*ycfL* mutants using *dadA* primers under the described conditions. 1 µg of each PCR reaction was loaded on 1% agarose gel.

Inspection of the metabolic fingerprints of wild-type BW25113 and the Δ*hinT* mutant revealed that while the Δ*hinT* mutant failed to grow on both D and L isomers of alanine, it did grow on serine and glycine. Based on these results, and the known *E. coli* alanine catabolic pathway, we hypothesized that the Δ*hinT* mutant was neither deficient in the amino acid transporter system for D,L-alanine, nor in alanine racemase, but most likely in D-amino acid dehydrogenase activity encoded by *dadA* (Fig. 3). The inability of the Δ*hinT* mutant to grow on D,L-alanine can be explained by either failure to induce transcription of the D-amino-acid dehydrogenase or the presence of a defective D-amino-acid dehydrogenase that is incapable of metabolizing D-alanine. To test these hypotheses, RT-PCR was performed using primers for *dadA* and equivalent amounts of mRNA obtained from wild-type BW25113 or the Δ*hinT* mutant grown in the presence of D,L-alanine. Results in Figure 4 show that equivalent amounts of *dadA* transcript were produced by either the wild-type strain or the Δ*hinT* mutant. To determine if the inability of the Δ*hinT* mutant to grow on D-alanine was due to a lack of DadA enzyme activity, membrane fractions were isolated and analyzed for D-amino acid dehydrogenase activity. Results showed that the Δ*hinT* mutant possessed 38-fold less D-amino acid dehydrogenase activity (<5 nmol/min/mg) relative to the wild-type BW25113 strain (190 nmol/min/mg).

### Phenotype rescue by *ecHinT*

**Figure 5:**
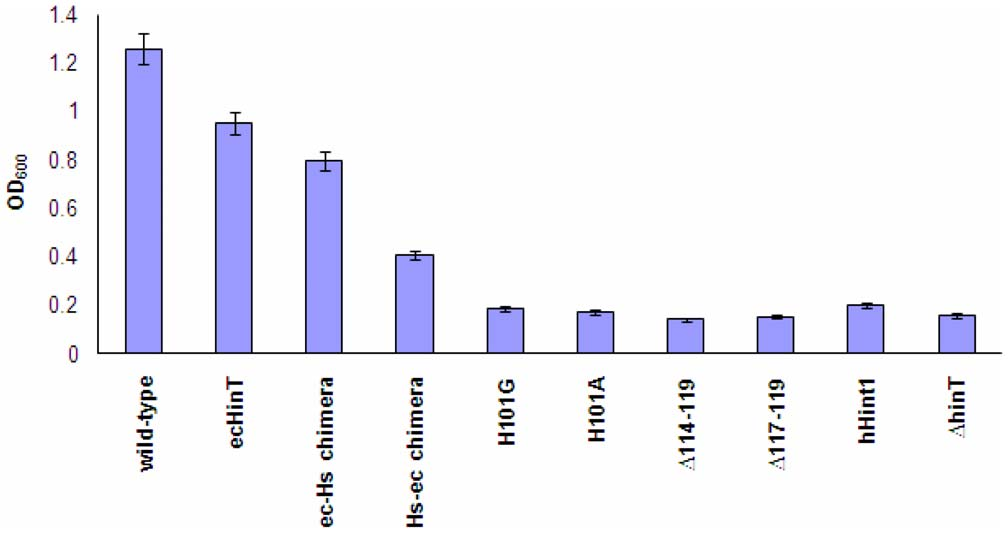
ecHinT structural and activity requirement for phenotype rescue. Each column represents the growth of the Δ*hinT* mutant that was transformed with a plasmid containing either *hinT*, a mutant *hinT*, *ec*/Hs chimera, Hs/*ec* chimera, or *hHint1*, except for the first column of wild-type *E. coli* BW25113. OD_600_ values were determined 48 h after inoculation into M9 medium in presence of 20 mM D,L-alanine.

Transformation of a plasmid encoding the wild-type *hinT* into the Δ*hinT* mutant (JW1089) allowed for growth on D,L-alanine to a level comparable to wild-type BW25113 grown under the same conditions (Fig. 5). This indicated that other mutations in strain JW1089 were not involved in the inability of the Δ*hinT* mutant to grow on D,L-alanine and that *hinT* was solely responsible for the observed phenotype.

A mutational approach was used to fully examine the relationship between ecHinT structure and activity, and DadA activity. Two active-site mutants were prepared and characterized as described previously [26]. The ecHinT H101 residue, which was previously shown to be required for enzymatic activity [26], was exchanged by site-directed mutagenesis into either alanine (H101A) or glycine (H101G). Enzymatic activity of these mutants were previously quantified by ^31^P-NMR using adenosine-5′- monophosphoramidate (AMP-NH_2_) as a substrate and the catalytic efficiency was found to be approximately 30,000-fold lower than that of wild-type ecHinT [26]. Plasmids encoding the catalytically impaired mutants of ecHinT, H101A or H101G, failed to complement the Δ*hinT* mutant for growth on D,L-alanine. This indicated that catalytically active ecHinT is necessary for DadA activity (Fig. 5).

Remote deletion mutations that reside in the C-terminus region of ecHinT have been prepared and characterized previously [17]. The Michaelis-Menten parameters for the deletion mutants, Δ114–119 and Δ117–119, have been shown to be within an order of magnitude of the values for wild-type ecHinT. However, phenotype rescue was not observed after over-expressing these proteins in Δ*hinT* mutant strain (Fig. 5).

### Design, synthesis and characterization of *ecHinT* inhibitor

**Figure 6:**
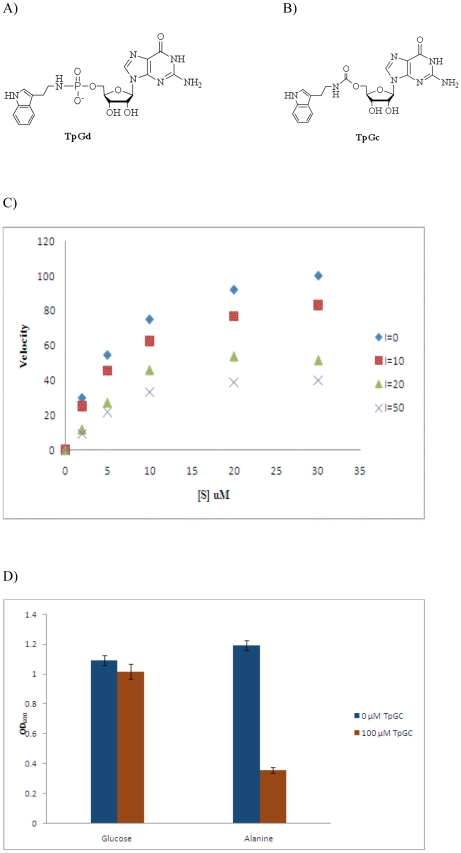
Inhibition of phenotype rescue by ecHinT inhibitor. A) The structure of tryptamine 5′-guanosine monophosphate (TpGd). B) The structure of tryptamine guanosine carbamate (TpGc). C) TpGc exhibits a non-competitive inhibition profile. D) Wild-type *E. coli* BW25113 was grown in M9 medium supplemented with either 20 mM glucose or 20 mM alanine in the presence (brown bar) or absence (blue bar) of 100 µM TpGC. OD_600_ values were determined after 48 h.

Tryptamine guanosine carbamate (TpGc) was prepared according to synthetic Scheme S1. The rationale for designing such an inhibitor was based on the structural activity relationship of Hint substrates that were reported previously [23]. The phosphoramidate substrate tryptamine 5′-guanosine monophosphate (TpGd) (Fig. 6A) was found to be an excellent substrate with a k_cat_ value of 4.0 s^−1^ and a k_cat_/K_m_ value of 7.0×10^5^ M^−1^s^−1^ [23]. It was hypothesized that substitution of the hydrolyzable phosphoramidate moiety with a carbamate linker (TpGc) would result in an inhibitor of ecHinT and not a substrate (Fig. 6B). In addition, the incorporation of guanosine would insure that off-target inhibition of Tryptophan tRNA synthetase would be minimized.

Using the previously described spectrophotometric phosphoramidase assay [23], the ecHinT K_i_ value for TpGc was determined. Although designed to be a competitive inhibitor, TpGc was found to exhibit apparent non-competitive inhibition of ecHinT with a K_i_ value of 42 µM (Fig. 6C). Consistent with our previous finding, that ecHinT catalytic activity is required for the phenotype, when compared to the growth of Δ*hinT E. coli* strain on D,L-alanine, cultures of wild-type BW25113 grown in the presence of D,L-alanine and 100 µM of TpGc exhibited a similarly diminished growth capacity (Fig. 6D).

## Discussion

The inducible *dadA* codes for the broad specificity bacterial D-amino-acid dehydrogenase. DadA catalyses the oxidation of D-amino acids, including alanine, into their corresponding ketoacids [27]. The bacterial D-amino-acid dehydrogenase is a heterodimer complex formed by a 45- and a 55-kDa subunit [28]. To avoid overproduction of reactive oxygen species that could damage cellular components, D-amino acid oxidation in bacteria is not coupled to O_2_ reduction and the two electrons from the substrate are received by FAD in the small subunit before being transferred to a sulfur-iron center in the large subunit [28]. Bacterial D-amino-acid dehydrogenase appears to have two main functions in bacteria. Initially, it permits the growth of bacteria when D-amino acids are the sole carbon, nitrogen, and energy source [29], [30]. Second, it prevents the accumulation of D-amino acids in cellular compartments, as some D-amino acid analogues have specific inhibitory effects on bacterial growth [31], [32]. In bacteria, D-amino acids are used to stabilize the peptidoglycan structure [33], and have recently been shown to regulate biofilm disorganization [34], as well as play a role in quorum sensing.

In this study, we report the first evidence of a regulatory relationship between the evolutionary conserved enzymes, DadA and ecHinT. Since transcripts encoding for *dadA* were produced in the *ΔhinT* mutant, our results suggested that D-amino acid dehydrogenase activity in *E. coli* is likely modulated by the interaction of ecHinT and DadA in a post-translational manner.

**Figure 7:**
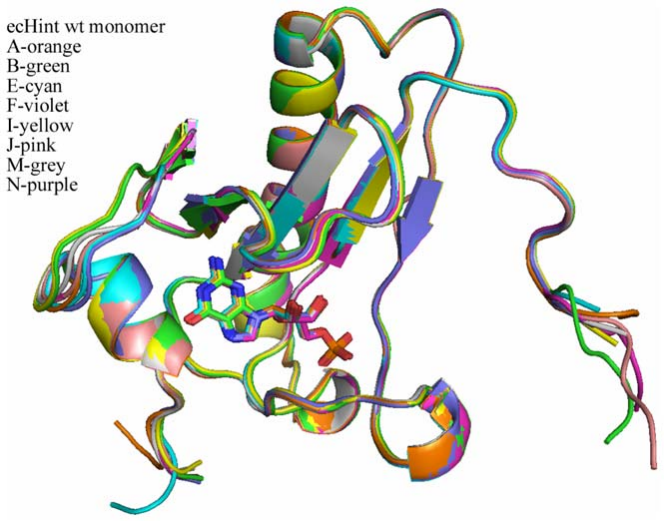
Superposition of the eight independent monomers observed in the structure of the wild-type ecHinT-GMP complex.

To probe structural elements of ecHinT that are determinant for the discovered phenotype, non-active-site deletion mutations that reside in the C-terminus region of ecHinT were prepared and characterized as described previously [17]. Recently, we investigated the C-terminal region of the ecHinT protein and found that this loop accommodates several conformational forms in the 1.3°A crystal structure (Fig. 7) [17]. We designed, and fully characterized, two deletion mutants of the ecHinT C-terminus in which three or six amino acids were deleted from the 11 amino acid C-terminal loop. The two deletion mutants were equivalent in their secondary structure to wild-type ecHinT and preserved the ability to form homodimers. The catalytic efficiencies of the deletion mutants were within one order of magnitude of the value determined for wild-type ecHinT. Despite the partial activity possessed by these mutants, both mutants failed to rescue *ΔhinT* growth on D,L-alanine.

Although both the N and C-terminal loops of ecHinT can adopt more than one conformation in the ecHinT-GMP complex structure [17], the overall fold of the structure is similar to hHint1 [17]. To investigate the possibility of phenotype rescue by hHint1, which is 48% homologous to ecHinT, the *E. coli* Δ*hinT* mutant was transformed with a plasmid encoding *hHint1* and the ability to grow on D,L-alanine examined. hHint1 failed to rescue the growth phenotype suggesting that certain structural characteristics, in addition to catalytic activity, govern the regulation of DadA activity by ecHinT. Based on our finding that the C-terminal loop of ecHinT plays a significant role in the observed relationship between ecHinT and DadA activity, the ability of two chimeric proteins to rescue Δ*hinT* cells was studied. The hHint1-ecHinT chimera (Hs/*ec*), in which the C-terminus of hHint1 was replaced with the ecHinT C-terminus, was unable to rescue the D-alanine growth defect exhibited by *E. coli* Δ*hinT* cells. In contrast, the ecHinT-hHint1 chimera (*ec*/Hs), in which the C-terminus of ecHinT was replaced with the hHint1 C-terminus, had only a slightly reduced ability to complement the phenotype when compared to wild-type ecHinT (Fig. 5).

Taken together, these results suggest that other inherent structural requirements, in addition to the C-terminus, are critical to the ecHinT-mediated DadA activation. Ongoing studies should shed light on the post-translational regulatory relationship between ecHinT and DadA and whether this regulation influences the localization, dimerization and/or degradation of DadA.

In summary, by using a series of mutagenic and chemical biological studies, ecHinT has been shown to be necessary for the growth of *E. coli* on D-alanine by regulating the catalytic activity of a key metabolizing enzyme, DadA. The role of ecHinT has been shown to be dependent on; the catalytic activity of ecHinT, components of its C-terminus domain and potentially unidentified structural factors. This marks only the second example of a direct relationship between the catalytic activity of a Hint protein and a physiological function [15]. Interestingly, the characteristics of ecHinT that appear necessary for the discovered phenotype are also required for the hydrolysis of LysU-generated lysyl-AMP by ecHinT [11], [17]. Whether this observation is a coincidence or evidence of a catalytic link between the interaction of echinT with LysRS and DadA remains to be determined.

## Supporting Information

Figure S1 **Sequence verification PCR (representative gel).** Forward primers were designed based on 5′ end of the gene of interest and the reverse primers were designed based on junction point between *Kan* resistance gene and the following gene in the operon. The length of each obtained product equals the size of the gene of interest plus the size of *Kan* resistance gene.
(DOC)

Figure S2 **Progress curves for ecHinT phosphoramidase activity in** ***E. coli*** **wild-type (WT) and ΔhinT cell-free lysates.**
(DOC)

Table S1 **Bacterial strains used in this study.**
(DOC)

Table S2 **Primers used for sequence verification PCR reaction.**
(DOC)

Scheme S1 **General synthetic scheme for TpGc.**
(DOC)
