# Supplementary material for: E. coli Histidine Triad Nucleotide Binding Protein 1 (ecHinT) Is a Catalytic Regulator of D-Alanine Dehydrogenase (DadA) Activity In Vivo
Source: PLoS One. 2011 Jul 6;6(7):e20897. doi: 10.1371/journal.pone.0020897 (PMC3130732; doi:10.1371/journal.pone.0020897)
Supplement: Figure S1 — Sequence verification PCR (representative gel). Forward primers were designed based on 5′ end of the gene of interest and the reverse primers were designed based on junction point between Kan resistance gene and the following gene in the operon. The length of each obtained product equals the size of the gene of interest plus the size of Kan resistance gene. (DOC) [file pone.0020897.s001.doc]

Figure S1: Sequence verification PCR (representative gel). Forward primers were designed based on 5’ end of the gene of interest and the reverse primers were designed based on junction point between *Kan* resistance gene and the following gene in the operon. The length of each obtained product equals the size of the gene of interest plus the size of *Kan* resistance gene.


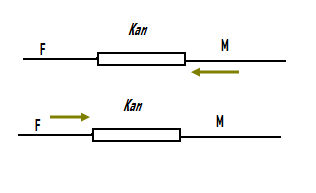


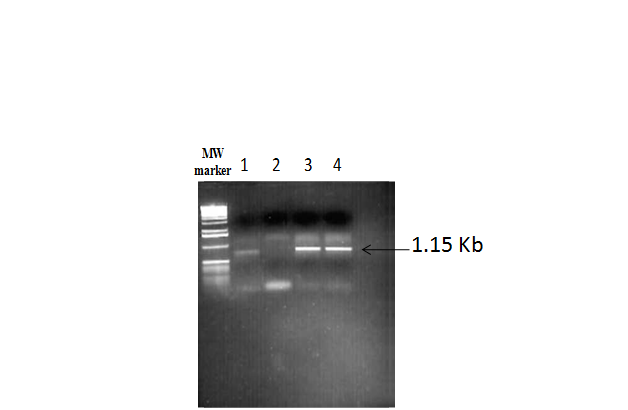


**Lane1: ∆*hinT* (annealing Temp 38 ºC)**

**Lane2: ∆*hinT* (annealing Temp 40 ºC)**

**Lane3: ∆*hinT* (annealing Temp 43 ºC)**

**Lane4: ∆*hinT* (annealing Temp 43 ºC)**

**Expected fragment size= 1.15**

***hinT* size(0.36Kb) + *Kan* gen (0.795)**
