# Supplementary material for: E. coli Histidine Triad Nucleotide Binding Protein 1 (ecHinT) Is a Catalytic Regulator of D-Alanine Dehydrogenase (DadA) Activity In Vivo
Source: PLoS One. 2011 Jul 6;6(7):e20897. doi: 10.1371/journal.pone.0020897 (PMC3130732; doi:10.1371/journal.pone.0020897)

Figure S2. Progress curves for ecHinT phosphoramidase activity in *E. coli* wild-type (WT) and ΔhinT cell-free lysates.


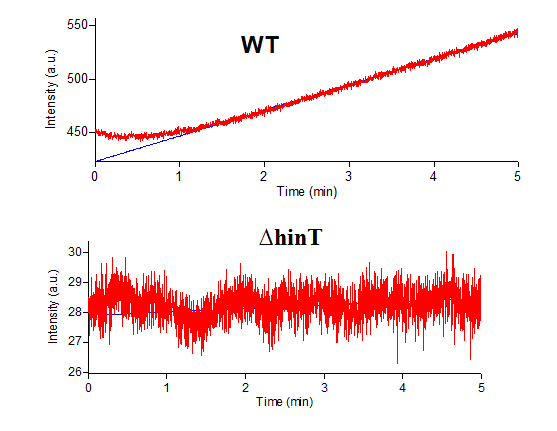

Supplement: Figure S2 — Progress curves for ecHinT phosphoramidase activity in E. coli wild-type (WT) and ΔhinT cell-free lysates. (DOC) [file pone.0020897.s002.doc]
