# Supplementary material for: E. coli Histidine Triad Nucleotide Binding Protein 1 (ecHinT) Is a Catalytic Regulator of D-Alanine Dehydrogenase (DadA) Activity In Vivo
Source: PLoS One. 2011 Jul 6;6(7):e20897. doi: 10.1371/journal.pone.0020897 (PMC3130732; doi:10.1371/journal.pone.0020897)
Supplement: Table S1 — Bacterial strains used in this study. (DOC) [file pone.0020897.s003.doc]

Table S1: Bacterial strains used in this study

| Gene deleted | Strain | Gene Size (Kb) | Gene Product |
| --- | --- | --- | --- |
| ***hinT*** | JW1089 | 0.360 | Purine nucleoside Phosphoramidase |
| ***ycfL*** | JW1090 | 0.378 | Predicted protein |
| ***ycfM*** | JW5157 | 0.642 | Predicted outer membrane lipoprotein |
| ***ycfN*** | JW1092 | 0. 825 | Thiamine kinase |
| ***nagZ*** | JW1093 | 1.026 | Beta-N-glucosaminidase |
| ***ycfP*** | JW5158 | 0.543 | Conserved protein |
