# Supplementary material for: E. coli Histidine Triad Nucleotide Binding Protein 1 (ecHinT) Is a Catalytic Regulator of D-Alanine Dehydrogenase (DadA) Activity In Vivo
Source: PLoS One. 2011 Jul 6;6(7):e20897. doi: 10.1371/journal.pone.0020897 (PMC3130732; doi:10.1371/journal.pone.0020897)
Supplement: Table S2 — Primers used for sequence verification PCR reaction. (DOC) [file pone.0020897.s004.doc]

Table S2: Primers used for sequence verification PCR reaction.

| Mutant | Forward primer | Reverse primer |
| --- | --- | --- |
| *∆hinT* | CGTCTACGGCACACCGCGTAA | GAATATAGTTTCTTCTGCCAC |
| *∆ycfL* | TGGCGCATAAAGGTCTGTAA | GGCGTAGCGACTCATTTTTGTC |
| *∆ycfM* | GGGGGCGCACAAAGTCAGACAA | GGGATTATTGCTGCGAATCGGC |
| *∆ycfN* | CTGGTCAGGTAAAGGTGCCGTT | GACATCCAACATTACTGGACCC |
| *∆ycfO* | CCTGGCGGCAGCTATTAATAAAA | CCGGACTGTTAGAGTCAAAACCG |
| *∆ycfP* | AAGCGTTCAAAACCCTCGGGTAA | CGCCGCCGACAATCACAATC |
