# Supplementary material for: E. coli Histidine Triad Nucleotide Binding Protein 1 (ecHinT) Is a Catalytic Regulator of D-Alanine Dehydrogenase (DadA) Activity In Vivo
Source: PLoS One. 2011 Jul 6;6(7):e20897. doi: 10.1371/journal.pone.0020897 (PMC3130732; doi:10.1371/journal.pone.0020897)
Supplement: Scheme S1 — General synthetic scheme for TpGc. (DOC) [file pone.0020897.s005.doc]

Scheme S1. General synthetic scheme for TpGc.
